# Adsorption of C.I. Natural Red 4 onto Spongin Skeleton of Marine Demosponge

**DOI:** 10.3390/ma8010096

**Published:** 2014-12-29

**Authors:** Małgorzata Norman, Przemysław Bartczak, Jakub Zdarta, Włodzimierz Tylus, Tomasz Szatkowski, Allison L. Stelling, Hermann Ehrlich, Teofil Jesionowski

**Affiliations:** 1 Institute of Chemical Technology and Engineering, Faculty of Chemical Technology, Poznan University of Technology, Berdychowo 4, Poznan 60965, Poland; E-Mails: malgorzata.norman@hotmail.com (M.N.); przemyslaw.bartczak88@gmail.com (P.B.); jakub_zdarta@wp.pl (J.Z.); tomasz.szatkowski88@gmail.com (T.S.); 2 Institute of Inorganic Technology and Mineral Fertilizers, Technical University of Wroclaw, Smoluchowskiego 25, Wroclaw 50372, Poland; E-Mail: wlodzimierz.tylus@pwr.edu.pl; 3 Department of Mechanical Engineering and Materials Science, Center for Materials Genomics, Duke University, 144 Hudson Hall, Durham, NC 27708, USA; E-Mail: antistokes@gmail.com; 4 Institute of Experimental Physics, Technische Universität Bergakademie Freiberg, Leipziger 23, Freiberg 09599, Germany; E-Mail: hermann.ehrlich@physik.tu-freiberg.de

**Keywords:** C.I. Natural Red 4, carmine, dye adsorption, kinetic model, marine sponge, spongin, *Hippospongia communis*

## Abstract

C.I. Natural Red 4 dye, also known as carmine or cochineal, was adsorbed onto the surface of spongin-based fibrous skeleton of *Hippospongia communis* marine demosponge for the first time. The influence of the initial concentration of dye, the contact time, and the pH of the solution on the adsorption process was investigated. The results presented here confirm the effectiveness of the proposed method for developing a novel dye/biopolymer hybrid material. The kinetics of the adsorption of carmine onto a marine sponge were also determined. The experimental data correspond directly to a pseudo-second-order model for adsorption kinetics (*r*^2^ = 0.979–0.999). The hybrid product was subjected to various types of analysis (FT-IR, Raman, ^13^C CP/MAS NMR, XPS) to investigate the nature of the interactions between the spongin (adsorbent) and the dye (the adsorbate). The dominant interactions between the dye and spongin were found to be hydrogen bonds and electrostatic effects. Combining the dye with a spongin support resulted with a novel hybrid material that is potentially attractive for bioactive applications and drug delivery systems.

## 1. Introduction

The synthetic dyes used in foodstuffs have relatively low production costs, high stability, and resistance to environmental conditions. This group of substances can be used to create a wide range of colors, as well as offering water solubility. They are also resistant to sudden changes in pH, temperature and light. In some cases, however, they may have a harmful effect on living organisms, and may contain undesirable additional substances [1,2]. Natural dyes used in foodstuffs, in turn, do not pose any risk to health, although they have weaker coloring properties and lower color intensity. They may also be sensitive to a number of factors: high temperature, changes in pH, and oxidants. Natural dyes are usually obtained by a process of extraction, purification, and concentration from plant or animal sources [3,4].

**Figure 1:**
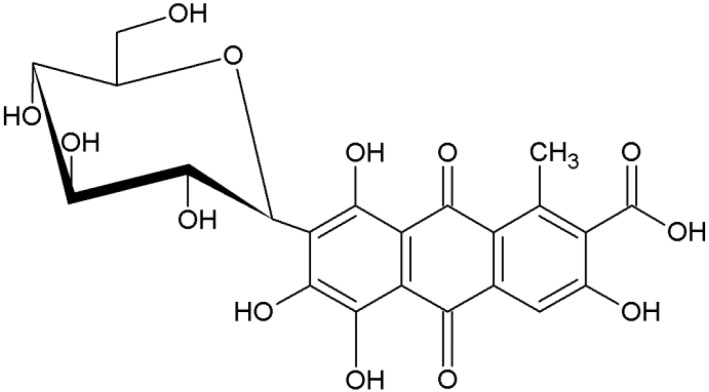
Structure of carminic acid.

Carmine (7-α-d-glucopyranosyl-9,10-dihydro-3,5,6,8-tetrahydroxy-1-methyl-9,10-dioxo-anthracene carboxylic acid), molecular weight 492 (g/mol), also called C.I. Natural Red 4 or cochineal, is a dark red dye obtained from dried and crushed insects from the Coccidae family (scientific name: *Dactylopius coccus*) [5,6]. Industrial carmine is obtained by mixing carminic acid with metal salts [7]. The structure of carminic acid is based on anthraquinone with multiple hydroxyl groups, a carboxyl group, and a glucose sugar unit side chain. The molecular structure of the dye is shown in Figure 1. This dye is susceptible to thermal decomposition and photodegradation, but exhibits relatively high chemical and biological stability [8].

As carmine is a harmless substance, it is used chiefly in the food, pharmaceuticals and cosmetics industries. It is also used as an indicator in analytical chemistry, and to a lesser extent in textiles and plastics [7]. Further uses include as a dye in microbiology, and for the modification of ion exchangers [9]. Due to the presence of its OH groups, it can form complexes with metal ions like U(VI), Th(IV), Mo(VI)) [10]. Carmine has also many biological applications: it can prevent coronary artery disease [11], it plays a role in treating Alzheimer’s disease [12], it exhibits cancer chemopreventive activity, [13] and is used in drug delivery products [14]. There exist a few publications concerning the adsorption of carmine or carminic acid, and they relate exclusively to inorganic adsorbents: TiO_2_ [15], glass beads [16], Amberlite XAD-16 resin [9], Ag nanoparticles [17], methacrylic acid (MAA) and 4-vinylpyridine [18], and multiwalled carbon nanotubes [19]. Carmine or carminic acid have also been combined with SiO_2_ sol-gel glass [8], hydrotalcite [20], and amorphous SiO_2_ [21].

Marine demosponges are representatives of the class Demospongiae that belong to the phylum Porifera [22]. The species *Hippospongia communis* also known as bath sponge, belongs to the Dictyoceratida order. Species of this order possess three-dimensional non-mineralized fibrous skeletons, which are composed mainly of the protein-like substance spongin. Spongin of Demosponges is very similar to collagen type XIII found in vertebrates which was confirmed by characteristic aminoacid composition [23,24]. Genomic and complementary DNA studies showed that spongin (similarly to collagen) contain the classic collagenous Gly-Xaa-Yaa motif where Hydroxyproline (Hyp) occupies any of the positions in the triplet motif, other than Gly (Glycine) position [25,26]. This biopolymer of still unknown chemical structure seems to be a naturally occurring hybrid between collagen and keratin-like proteins that contains sulfur, bromine and iodine [27,28,29,30,31]. Because of its unique physico-chemical, structural, and mechanical properties [32] spongin-based skeletons of bath sponges has been broadly used since ancient times in household use and medicine [33]. Nowadays, their biocompatibility [34] and specific arrangement of structural elements like pores, struts and channels offers model scaffolds for tissue engineering [35,36]. Spongin-containing marine sponges, including Mediterranean *H. communis*, are examples of renewable resources due to their ability to be cultivated under marine ranching conditions [37,38]. This property enhances the biomimetic potential of bath sponges as organisms, and that of spongin as a specific biological material. In contrast to the attempts to dye bath sponges with synthetic dyes, their ability to adsorb natural dyes is still not studied.

Thus, Cohn in his patent [39] reported as follows: “It has been suggested, as disclosed in the English Patent to Asher 14,866 of 19 July 1905, that sponges of some unidentified type could be dyed when treated first with a metallic mordant at some unidentified temperature and then dyed in an alizarine bath at temperatures of 70–80 °C (158–176 °F). The primary objection to sponges dyed in accordance with the suggestions in the Asher patent is that the colors are not fast, and the resulting so-called sponge bleeds when wetted with warm water. Further, mordants of the type in general use in 1905, such as the basic aluminum sulfates suggested, in order to be effective must necessarily be heated at temperatures approaching the boiling point (even if the boiling point is not actually reached). Sponges dyed by the method disclosed in this English patent do shrivel up despite the claim in the patent that shriveling is avoided when the alizarine lakes are maintained at temperatures not exceeding the 70–80 °C. However, a more serious objection than simply that the sponge becomes shriveled and cannot retain the color of the lake in which it was dyed, is that it is otherwise deleteriously affected in its physical characteristics—for instance, it loses to a large extent its elasticity, or spring, or “life”. Any temperature as high as 70–80 °C appears to destroy or at least partially close the inhalant pores, the canals, the apoyles, and the oscules, and thus interferes with the water flow through the sponge’s passageways.

It has been found that bleached sponges dye much more readily than unbleached ones. Bleached sponges also require about one-half as much color as unbleached sponges do. The bleaching of the sponge apparently doubles its color absorbing qualities. Bleaching, particularly with the permanganate method proposed, gives the sponge aseptic properties and the developers used in Step I act additionally as preservatives to prevent bacteria and mold growth [39].

To our best knowledge, there are no reports to date concerning the adsorption of natural dyes using sponginous skeletons of marine sponges as a support. The aim of the present study is to obtain interesting dye/biopolymer hybrid materials. Combining the dye with a support improves its bioavailability and its resistance to chemical and thermal degradation. This enhanced stability creates opens the door for future applications, which may include creating a biocompatible material used in drug delivery.

## 2. Results and Discussion

### 2.1. Spectrophotometric Investigation

Absorption spectra (400–900 nm) were obtained for carmine in water at different pH values. Over the analyzed pH range (3–11) the absorption maxima varied only slightly. The color of the C.I. Natural Red 4 water solution showed marginal variation, as it exists in several forms [17]. Measurements of the absorbance of C.I. Natural Red 4in a solution with pH = 7 over the full visible light range showed a maximum absorbance at 513 nm. The absorption spectrum of carmine in water undergoes a red shift (shift to a longer wavelength absorption) upon addition of metal salts [40]. Based on the literature data, for pure carminic acid this value equals 493 nm.

### 2.2. Effect of Contact Time and Dye Concentration

**Figure 2:**
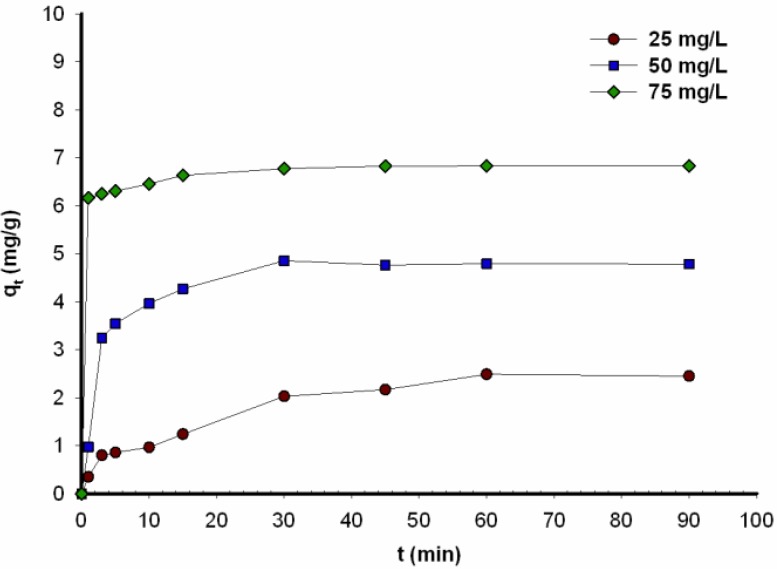
Adsorption capacity for C.I. Natural Red 4 onto *H. communis* sponge skeleton, as a function of time (results obtained in pH = 7).

Figure 2 shows the effect of carmine concentration on the amount of the dye adsorbed (*q_t_*) on the *H. communis* spongin scaffold, plotted against time.

The highest value of *q_t_* was obtained after reacting for 90 min. The adsorption capacities for 25, 50 and 75 mg/L of C.I. Natural Red 4 onto the marine sponge skeleton were 2.45, 4.79 and 6.84, respectively.

The efficiency of C.I. Natural Red 4 adsorption decreased as the initial concentration increased, even though the quantity of dye adsorbed per unit mass of adsorbate increased. This is linked to the quantity of molecules of the dye adsorbed on the surface of the support: the smaller the dye concentration, the more molecules present in the solution can become bound to the adsorbent. An increase in concentration leads to saturation of the active sites of the support, which reduces the efficiency of the process; because in effect a significant number of dye molecules are not adsorbed [41]. Adsorption is reduced due to the lack of sufficient available open sites to adsorb high initial concentrations of the dye [11,12,13,14,15,16]. This situation is caused by the mass transfer driving force, which increases when the initial concentration is increased, resulting in higher adsorption of dyes [42,43]. Various studies have confirmed that adsorption capacity increases as the dye concentration increases [44,45]. As can be seen from Figure 2, the quantity of dye adsorbed rose very rapidly in the course of the first few minutes of the process, for all tested concentrations of dye in solution. Similar effects were observed when carmine was absorbed onto glass beads [16]. The contact time needed for C.I. Natural Red 4 (in every initial concentration) to reach equilibrium was around 30 min. The quantity of carmine adsorbed on marine sponges increases with time, and reaches a constant value beyond which no more dye is removed from the solution. At this point, the quantity of dye desorbing from the marine sponge skeleton is in a state of dynamic equilibrium with the quantity being adsorbed onto it. This observation can be explained by the theory that diffusion onto the external surface of the adsorbent was followed by diffusion into the intra-particle matrix to attain equilibrium [46].

### 2.3. Effect of pH

The manner in which the pH of the environment affects the efficiency of adsorption of the dye from solution was also examined. The process was carried out at pH = 3, 5, 7 and 9, for initial dye concentrations of 25, 50 and 75 mg/L. Adsorption tests were performed over 30 min.

It was observed that an increase in the acidity of the solution increases the efficiency of the adsorption of C.I. Natural Red 4. The quantity of dye adsorbed from a 25 mg/L solution increases from 1.42 mg/g (56.7%) at pH = 7 to 2.45 mg/g (98.1%) at pH = 3. In a basic solution, however, the efficiency of the adsorption process is zero. The same patterns were observed for the other carmine concentrations. This is linked to the protonation of the neutral –NH_2_ amine groups in the protein scaffold to form –NH_3_^+^ cationic groups. Under these conditions the process of adsorption of the dye occurs via electrostatic interactions. An increase in the pH leads to deprotonation of –NH_3_^+^ groups, and in effect only hydrogen bonds [47] are formed between the support and the dye, reducing the efficiency of adsorption. Similar adsorption behavior as the pH is varied has been reported in the literature for compounds containing NH_2_ groups [42,44,48]. The results are presented in Table 1.

**Table 1:** Effect of pH on adsorption capacity for C.I. Natural Red 4 onto marine sponge.

pH	Dye concentration (mg/L)		
	25	50	75
	Experimental *q_t_* (mg/g)		
3	2.45	5.00	7.50
5	1.50	3.62	6.71
7	1.42	3.42	5.99
9	0.00	0.00	0.00

A similar range of pH values was used during carminic acid impregnation of the resin Amberlite XAD-16. A continuous decrease in impregnation efficiency at both pH > 3 and pH < 3 was observed. The lowest impregnation efficiency of 89% was at pH = 8 [9]. However, the required time needed for completing the impregnating process was found to be at least 3 h. A similar effect of pH on carminic acid adsorption is also described in [17].

### 2.4. Desorption Test

Desorption tests for samples containing C.I. Natural Red 4 were carried out at different pH values (7 and 9). In contrast with the adsorption tests performed at varying pH, the results in this case were not affected by the acidity of the environment. The efficiency of the process was 13.4% at pH = 7, and 12.8% at pH = 9. It was observed, however, that the desorption percentage was greater when the dye was washed from samples that contained a greater quantity of dye following the adsorption process. In the case of marine sponge skeleton pieces containing 4.54 mg/g of C.I. Natural Red 4 the desorption percentage was 18.8%, while from samples containing 2.27 mg/g the percentage was 4.7%.

### 2.5. Kinetic Analysis

To investigate the kinetics of the adsorption process, pseudo-first-order (PFO) and pseudo-second-order (PSO) models were used. These investigations make it possible to describe the controlling mechanism of the adsorption process.

A pseudo-first-order equation is:

(1) $$\log\left(q_{\text{e}}-q_{t}\right)=\log\left(q_{\text{e}}\right)-\frac{k_{1}}{2.303}\cdot t$$

where *q_t_* and *q*_e_ (mg/g) are the quantities of dye adsorbed at time *t* (min) and at equilibrium, and *k*_1_ (1/min) is the rate constant of pseudo-first-order sorption. The pseudo-first-order model refers to an adsorption process in which sorption proceeds by diffusion through a boundary.

A pseudo-second-order equation is:

(2) $$\frac{t}{q_{t}}=\frac{1}{k_{2}q_{\text{e}}^{2}}+\frac{1}{q_{\text{e}}}\cdot t$$

where *k*_2_ (g/mg·min) is the pseudo-second-order rate constant. When the adsorption process proceeds according to a pseudo-second-order model, the limiting step may be chemical adsorption involving valent forces through the sharing or exchange of electrons between the sorbent and adsorbate [49]. In [16], the adsorption kinetics of C.I. Natural Red 4 were described using the kinetic approximations proposed by McKay and Boyd. While adsorption is possible as a result of interaction of the functional groups of carmine and glass beads, its rate is controlled by film and particle diffusion.

**Figure 3:**
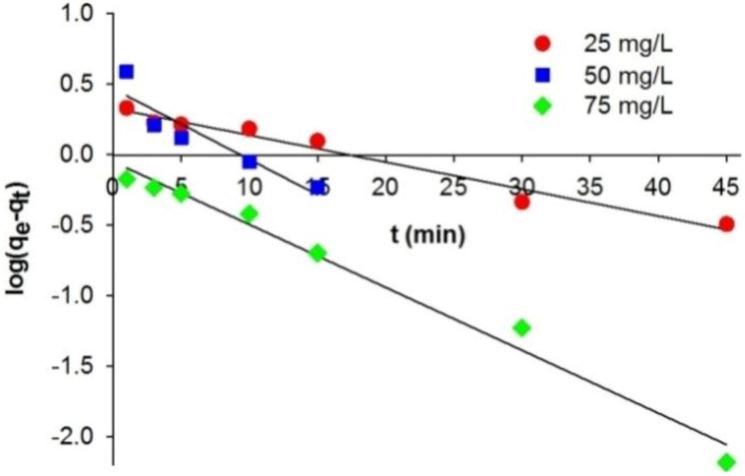
Pseudo-first-order kinetic fit for adsorption of C.I. Natural Red 4 onto *H. communis* sponge skeleton.

In this study, the kinetics of the adsorption process were described using different pseudo-first-order and pseudo-second-order models. The equilibrium adsorption capacity (*q*_e_) and adsorption rate constant (*k*_1_) (Table 2) were computed experimentally from a plot of log(*q*_e_ *– q_t_*) against *t* (Figure 3).

**Figure 4:**
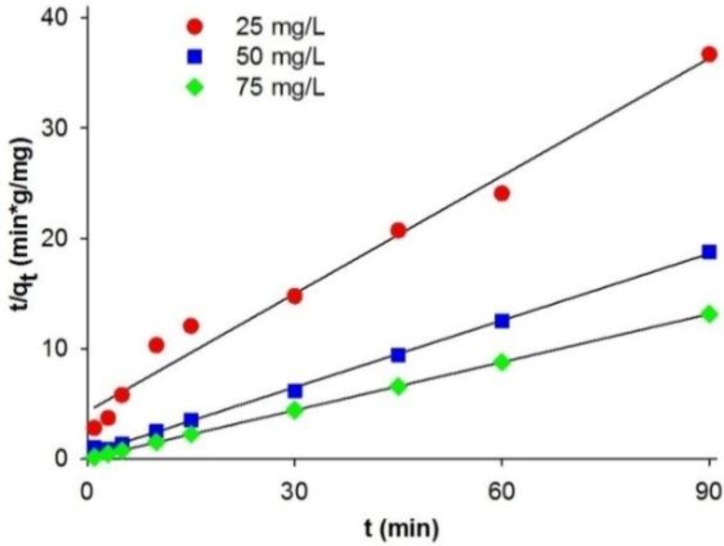
Pseudo-second-order kinetic fit for adsorption of C.I. Natural Red 4 onto *H. communis* sponge skeleton.

The coefficient of correlation (*r*^2^) obtained when a pseudo-first-order kinetic model is used to describe the adsorption of C.I. Natural Red 4 (in concentrations of 25–75 mg/L) lies in the range 0.855–0.986. The values of adsorption capacity (*q*_e,cal_) computed from the pseudo-first-order kinetic model deviated significantly from the experimental capacities (*q*_e,exp_). This indicates that the pseudo-first-order kinetic model does not fit well to the experimental data. A significantly better model describing the kinetics of adsorption of C.I. Natural Red 4 onto the marine sponge is the pseudo-second-order kinetic model (Figure 4). The pseudo-second-order model *k*_1_ value is lower than *k*_2_, indicating that the pseudo-second-order equation better describes the adsorption process.

**Table 2:** Pseudo-first-order and pseudo-second-order kinetic parameters and coefficient of determination for adsorption of C.I. Natural Red 4 onto marine sponge.

Type of kinetics	Parameters		Concentration of dye (mg/L)		
	Symbol	Units	25	50	75
	*q*_e,exp_	mg/g	2.492	4.858	6.836
Pseudo-first-order	*q*_e,cal_	mg/g	3.799	4.229	7.232
	*k*_1_	1/min	0.044	0.116	0.100
	*r*^2^	–	0.970	0.855	0.986
Pseudo-second-order	*q*_e.cal_	mg/g	2.810	4.951	6.869
	*k*_2_	1/min	0.029	0.092	0.382
	*r*^2^	–	0.979	0.999	0.999
	*h*	mg/g min	0.232	2.246	18.023

### 2.6. Adsorption Isotherms

Adsorption isotherm is a graphical representation indicating the relation between the mass of the adsorbed dye per mass of the used adsorbent, and liquid phase of dye at equilibrium concentration. Based on the experimental data adsorption isotherms were determined based using the Freundlich [42] and Langmuir [41] models.

**Figure 5:**
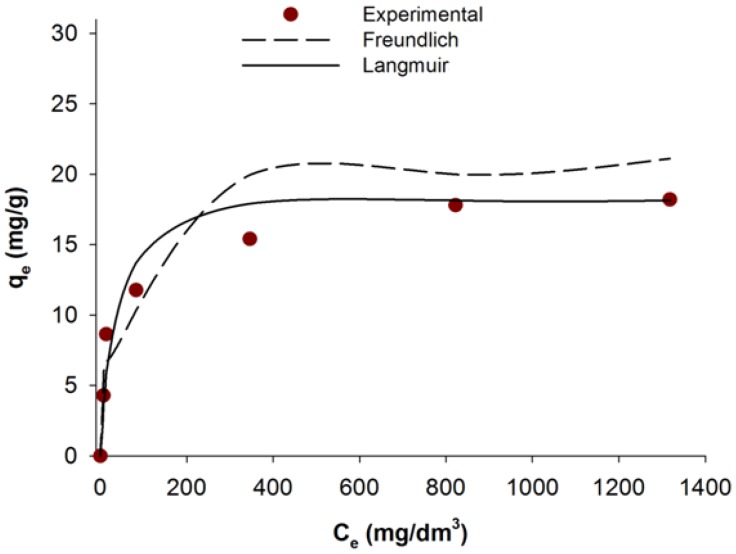
Fitting of the Langmuir and Freundlich isotherm models to equilibrium results of C.I. Natural Red 4 adsorbed onto marine sponge skeleton.

The plot of (*q*_e_) *versus* (*C*_e_) for the adsorption isotherms of C.I. Natural Red 4 onto the Demosponge skeleton is presented in Figure 5. Table 3 shows the parameters for the Freundlich and Langmuir isotherms.

The Freundlich equation is given as:

(3) $$q_{\text{e}}=K_{\text{F}}\cdot C_{\text{e}}^{\frac{1}{n}}$$

where *C*_e_ is the equilibrium concentration of the dye (mg/L), *q*_e_ is the quantity of the adsorbed dye per mass of adsorbent (mg/g), and *K*_F_ (mg/g) and *n* are the Freundlich constants. The *K*_F_ and *n* values can be estimated from the intercept and slope of a linear plot of log*q*_e_ *versus* log*C*_e_.

**Table 3:** Freundlich and Langmuir isotherms constants for C.I. Natural Red 4 adsorbed onto marine sponge skeleton.

Langmuir parameters			Freundlich parameters		
*R*^2^	*q*_m_ (mg/g)	*b* (L/mg)	*R*^2^	*K*_F_ (mg/g)	*n*
0.995	18.55	0.034	0.877	3.601	4.195

The value *n* determines the degree of nonlinearity between the solution concentration and adsorption: a value *n* < 1 indicates a normal isotherm, while *n* > 1 indicates a cooperative adsorption. The value *n* computed from Freundlich’s equation for the adsorption of C.I. Natural Red 4 onto the marine sponge skeleton is equal to 4.195.

The equation of a non-linear Langmuir isotherm model takes the following form:

(4) $$q_{\text{e}}=\frac{q_{\text{m}}\cdot b\cdot C_{\text{e}}}{1+b\cdot C_{\text{e}}}$$

where *C*_e_ is the equilibrium concentration in the solution (mg/L), *q*_m_ is the maximum adsorption capacity and *b* is the Langmuir constant (L/mg), calculated from the intercepts and slopes of linear plots of *C*_e_*/q*_e_ *versus C*_e_.

The sorption capacity calculated using the Langmuir model was equal to 18.55 mg/g. Comparing the isotherms’ parameters it can be concluded that the experimental data definitely resemble the Langmuir model, which is borne out by the high correlation coefficient (*R*^2^ = 0.995).

### 2.7. FT-IR

To confirm the effectiveness of adsorption of C.I. Natural Red 4 onto spongin fibers, FT-IR spectra of the products were taken to check for the presence of characteristic functional groups. Detailed investigations were performed for the *H. communis* sponge skeleton and C.I. Natural Red 4. Additional measurements were made for the dye/spongin hybrid material obtained from an initial dye concentration of 50 mg/L, and a reaction time of 30 min. Details of the bands present in the spectra, with their wavenumbers and band assignments, are given in Table 4.

**Table 4:** FT-IR characteristic wavelengths for C.I. C.I. Natural Red 4, marine sponge and hybrid material (dye solution 50 mg/L, contact time 30 min, pH = 7).

C.I. Natural Red 4	*Hippospongia communis skeleton*	Dye/Biopolymer hybrid material	Vibrational assignment
3400	3410	3415	–OH stretching
–	3300	3310	–NH stretching
2930	2930	2930	–CH_2_, –CH_3_ stretching
1650	1630	1655	C=O stretching
1560	–	1560	C=C_Ar_ stretching
–	1520	1525	–NH deformational
–	1460	1460	–CH scissors
1400	1400	1405	–OH stretching
–	1250	1250	C–N stretching
1080	1080	1075	C–O–C stretching
1020	1020	1020	C–O stretching
900	–	907	–OH bending
660	–	660	–CH_Ar_ deformational
520	–	525	

The spectrum for the adsorbent (*Hippospongia communis*) displays signals indicating the presence of –NH bonds (at 3300 cm^−1^ and 1520 cm^−1^) and C–N bonds (at wavenumber 1520 cm^−1^), which are part of the proteinaceous (spongin) skeleton of the sponges. The vibrations generating these bands occur only in the structure of the spongin; they are not observed in the dye molecule.

The bands at 1560 cm^−1^ (C=C_Ar_ stretching vibrations), 660 cm^−1^ and 520 cm^−1^ (–CH_Ar_ deformational vibrations) are found as original signals, and are only observed in C.I. Natural Red 4. There is also a clear signal at wavenumber 900 cm^−1^ due to bending vibrations of OH groups from carboxyl group. All of these data are in agreement with the literature [6,15,19].

The spectrum of the dye/biopolymer hybrid material reveals the presence of signals characteristic for both the adsorbent and carmine. However, the maxima are shifted in the direction of higher wavenumber values for the stretching vibrations of hydroxyl groups (3415 cm^−1^ and 1405 cm^−1^) and the stretching vibrations of C=O groups (1655 cm^−1^). The same occurs for the bands generated by stretching and deformational vibrations of –NH groups in the sponges (at 3310 cm^−1^ and 1525 cm^−1^) and the bending vibrations of carboxylic–OH groups in the dye (at 907 cm^−1^). Apart from the shift in the maxima, certain signals are found to be more intense in the spectrum of the hybrid material. This is particularly visible in the case of the bands assigned to hydroxyl groups (3415 cm^−1^ and 1405 cm^−1^), stretching vibrations from –CH_2_ and –CH_3_ groups (2930 cm^−1^), and stretching vibrations due to C=O (1630 cm^−1^). This is due to the fact that these functional groups are present in both starting materials. A further factor may be the mechanism of adsorption of the dye on the sponge surface (the formation of hydrogen bonds between their surface groups, which are responsible for the observed chemical shifts) [50].

**Figure 6:**
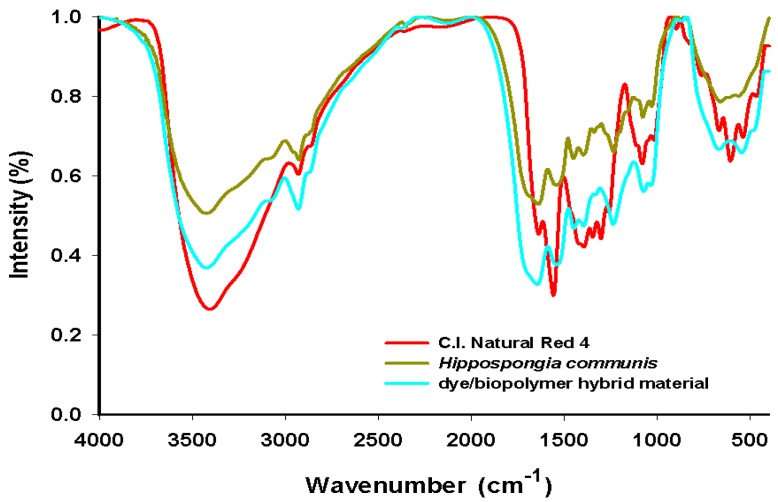
FT-IR spectra of C.I. Natural Red 4, marine sponge and hybrid material (dye solution 50 mg/L, contact time 30 min, pH = 7).

The results are presented in Figure 6.

### 2.8. XPS

The chemical structures of carmine, marine sponge skeleton and the dye/biopolymer hybrid were also studied by XPS spectroscopy. This was used to determine the relative quantities of dye adsorbed on the spongin biopolymer surface.

**Figure 7:**
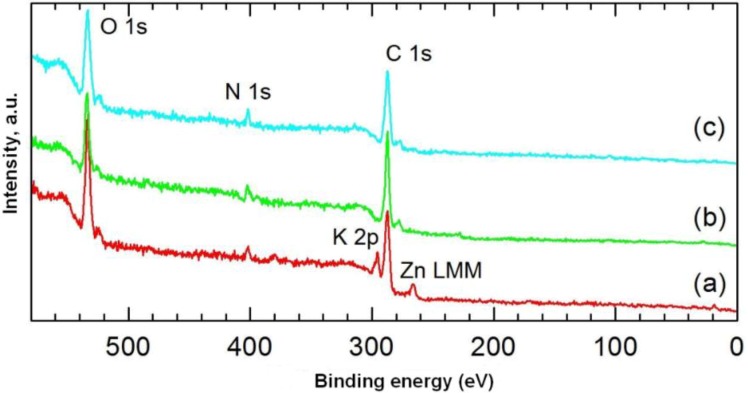
XPS spectra of: (**a**) C.I. Natural Red 4; (**b**) *H. communis* sponge skeleton; (**c**) hybrid material (obtained from 75 mg/L dye solution, contact time 30 min, pH = 3).

**Figure 8:**
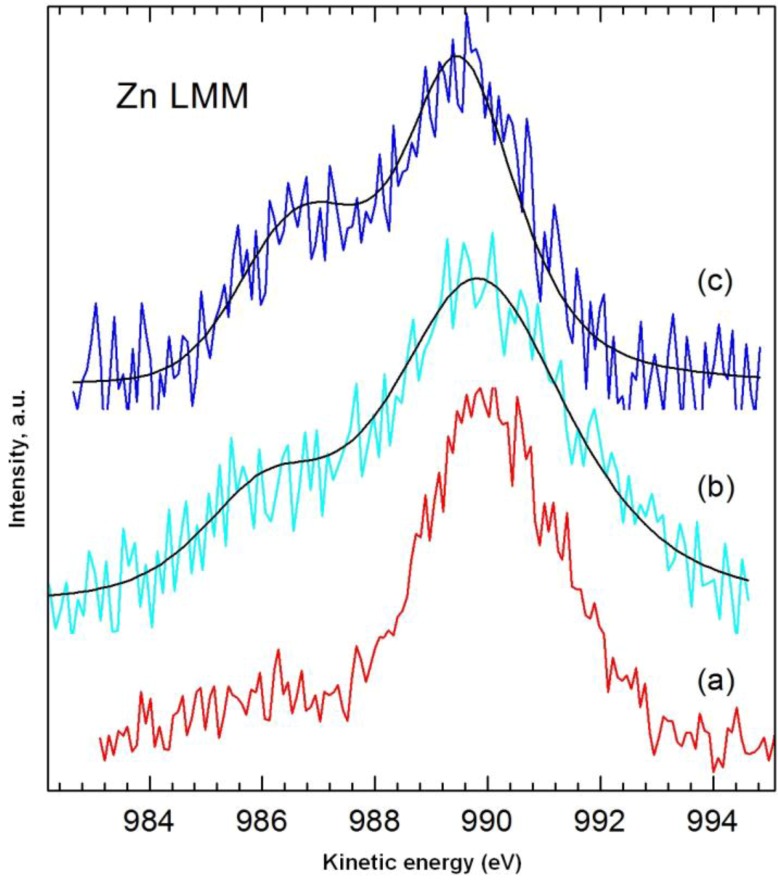
High-resolution XPS spectrum of Zn LMM photoelectrons: (**a**) dye; (**b**) sample 1 (dye solution 25 mg/L, contact time 30 min, pH = 3); (**c**) sample 3 (dye solution 75 mg/L, contact time 30 min, pH = 3).

Figure 7 shows XPS spectra of the dye, the spongin, and a selected dye/biopolymer material. Table 5 contains the results of quantitative analysis of the samples. Due to the high similarity of the high-resolution spectra for the main components C 1s, O 1s and N 1s in the support and the dye, they could not be used as a basis for determining the adsorbed quantity of dye. In addition, no changes were found in the bond energies of the aforementioned components following impregnation of the support with dye. Among the elements potassium, zinc, silicon and sulphur identified in the dye (Table 5), only zinc was detected on the surface of the dye/biopolymer hybrid. The quantity of this element rose from 0.16% to 0.31% in samples 1–3 (Table 6), in accordance with the sequence of increasing concentration of dye in the initial solution. The quantity of zinc on the surface of the samples was determined using the main Auger Zn LMM line of photoelectrons (Figure 8), in view of their superior quality compared with the line Zn 2p_3/2_ (at such low surface concentrations of zinc). The value of the modified Auger α’ parameter for Zn LMM and Zn 2p_3/2_ electrons for the pure dye and the tested samples was approximately 2010 eV, indicating the presence of ZnO.

**Table 5:** Surface composition and relative concentration of elements obtained by XPS analysis of the marine sponge skeleton, C.I. Natural Red 4, and selected hybrid materials.

Sample	C	N	O	K	S	Zn	Si
*Hippospongia communis*	72.54	5.25	22.21	–	–	–	–
C.I. Natural Red 4	57.90	4.17	31.43	3.60	0.83	2.05	–
Sample 1 (25 mg/L, 30 min, pH = 3)	66.63	7.37	24.44	–	–	0.16	1.40
Sample 2 (50 mg/L, 30 min, pH = 3)	67.59	6.08	24.75	–	–	0.18	1.40
Sample 3 (75 mg/L, 30 min, pH = 3)	68.52	4.85	26.32	–	–	0.31	–

**Table 6:** Zinc content in selected dye/biopolymer samples (dye solution 1:25 mg/L; 2:50 mg/L; 3:75 mg/L; contact time 30 min, pH = 3).

Sample	1	2	3
Zn, % at.	0.16	0.18	0.31
Zn:N	0.022	0.030	0.064

Clearer confirmation of the correlation between the concentration of dye in the initial solution and its content in samples 1–3 is provided by analysis of the Zn:N ratio, which increased from 0.022 in sample 1 to 0.062 in sample 3. The relatively greater increase found for the Zn:N ratio than for the absolute content of Zn results from higher coverage of the surface by the dye. It should be noted that the greater part of the nitrogen recorded in samples 1–3 came from the biopolymer (>90%, estimated from the decrease in Zn content in the samples compared with the pure dye); also the N 1s bond energies for samples 1–3 and the biopolymer were identical at 399.75 eV, compared with 399.30 eV for C.I. Natural Red 4.

### 2.9. NMR

The effectiveness of the modification was also verified by means of ^13^C CP/MAS NMR. The results proved that the carmine/spongin interactions are of a chemical nature. Figure 8 shows a ^13^C CP/MAS NMR spectrum of C.I. Natural Red 4, spongin and the obtained hybrid material. Attribution of peaks for the dye was made according to [21,51]. The most intense signals, occurring in the range 60–80 ppm, come from the glucose residue, and the signal at 20.4 ppm is from a CH_3_ group. The chemical shifts observed above 100 ppm are attributed to aromatic carbons (C=C bonds), and that at δ = 178.2 ppm to the carbon of a carboxyl group. The ^13^C CP/MAS NMR spectrum of *H. communis* spongin indicates the presence of aliphatic carbon (saturated alkanes), as well as carbon bonded to nitrogen (C–NR_2_) and to oxygen (C–OH and C–OR) with signals in the 20–80 ppm range. There is also a marked signal at δ = 174.3, which is characteristic of carbon occurring in a carboxyl group or its derivatives [30,52]. Marine sponge spongin has an inexact chemical structure, where each resonance represents not just one but a range of chemical environments. Due to the lack of NMR data, the attachment position was not elucidated but only proposed. However, comparing the spectrum obtained for the spongin with that of collagen [53], many similarities are observed, indicating the high degree of similarity of their structures.

**Figure 9:**
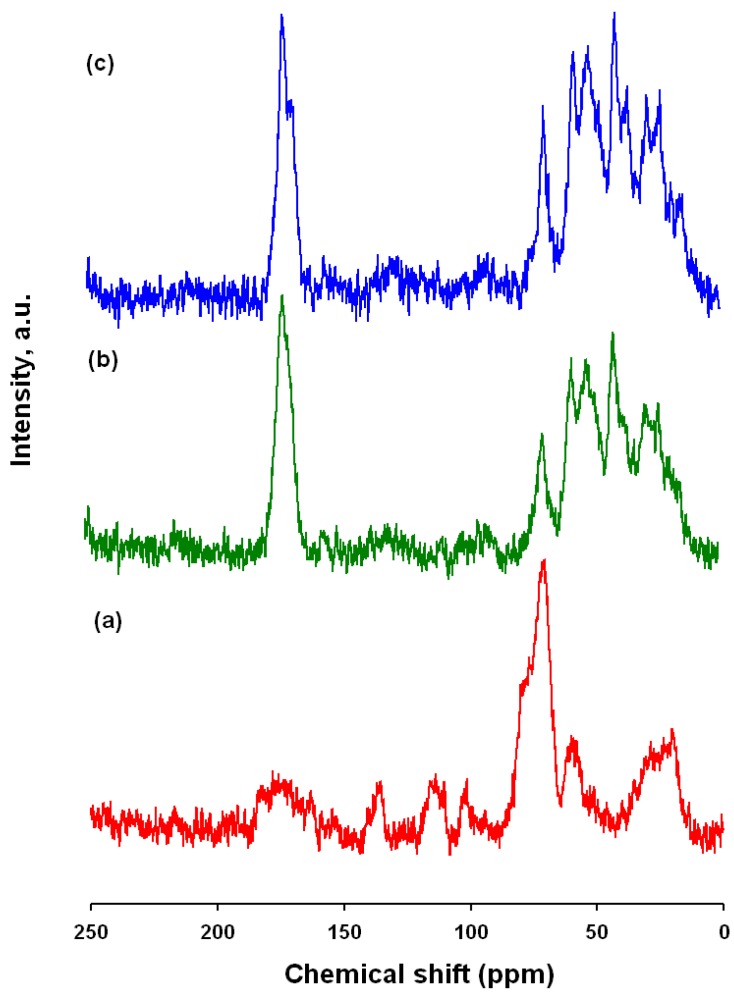
^13^C CP/MAS NMR spectra of: (**a**) C.I. Natural Red 4; (**b**) hybrid material (dye solution 75 mg/L; contact time 30 min, pH = 3); (**c**) *H. communis* spongin.

The spectrum for one of the dye/biopolymer materials is shown in Figure 9. It contains a number of signals which are not seen in the spectrum of the adsorbent: δ = 170.9, 69.8, 49.8, 38.5, 21.5 ppm. In the range 100–150 ppm, as the quantity of adsorbed dye increases, peaks corresponding to aromatic carbon become visible. Moreover, comparing the spectra of the hybrid product and marine sponge skeleton (taken as a reference sample), changes in the intensities, positions and widths of other signals are observed.

Unfortunately, some resonances are difficult to observe because of the low signal/noise ratio.

### 2.10. Raman Spectroscopy

**Figure 10:**
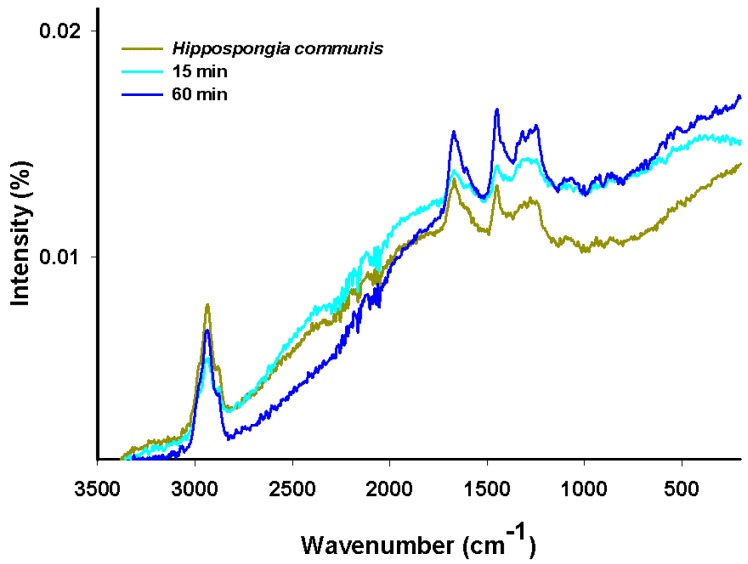
Raman spectra of *H. communis* spongin and selected hybrid materials (dye solution 100 mg/L, pH = 7, contact time 15 and 60 min).

The Raman spectra of carmine, marine sponge skeleton and dye/biopolymer material are shown in Figure 10. In the Raman spectroscopy results, as in the case of NMR spectra, the spectrum for the sponge material (spongin) is similar to that of collagen [54].

The bands at 2883 cm^−1^ and 2938 cm^−1^ can be attributed to the weak stretching mode of OH and medium-strong asymmetric stretching of CH_3_. The signal at 1671 cm^−1^ corresponds to the weak-medium stretching mode of C=O, that at 1448 cm^−1^ to weak N–H bending, and that at 1281 cm^−1^ to weak-medium stretching of C–N. The signals between 1100 cm^−1^ and 1000 cm^−1^ can be attributed to weak asymmetric stretching of C–O–C. Some additional signals, originating from CH and CH_3_ in the glucose residue of carmine, are observed in two spectral regions (1350–1050 cm^−1^ and 880–680 cm^−1^) [17,55]. The increase in the intensity of signals at 1670 cm^−1^and 1452 cm^−1^, and the appearance of peaks at around 550 cm^−1^ (skeletal vibration), are associated with stretching vibrations in benzene rings in the dye.

In the case of the dye/biopolymer hybrid material, the results of Raman spectra analysis were similar to those for the marine sponge. Analysis of the spectra did not reveal any new bands; however, the bands’ intensity changed as a consequence of overlapping of the bands characteristic of marine sponge and dye. We conclude that the dye interacts by hydrogen bonding with the hydroxyl and carbonyl groups of spongin.

### 2.11. Thermal Stability

Thermal analysis is the main method for determining the thermal properties of chemical substances. The results of these measurements, which give information about the thermal stability of a substance, are among the most important parameters determining the range of potential applications for materials.

**Figure 11:**
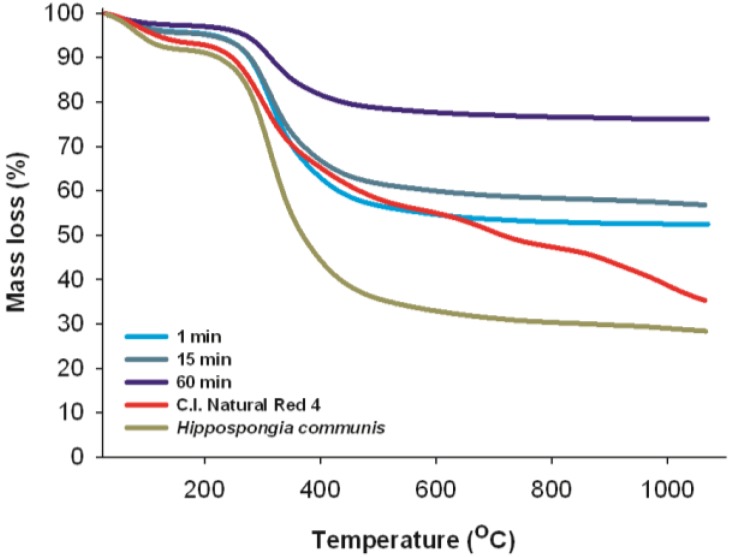
Thermal analysis of C.I. Natural Red 4, *H. communis* spongin and selected hybrid materials (dye solution 25 mg/L, pH = 7, reaction time 1, 15 and 30 min).

The thermal decomposition profiles of marine sponge skeleton and C.I. Natural Red 4 are shown in Figure 11. As mentioned above, both sponge skeletons consist of the protein-like spongin. It is observed from the TG curve that the thermal degradation of *H. communis* spongin takes place in two stages. The first stage, in the range 80–110 °C, is associated with the evaporation of water. The second stage, involving considerable mass loss (60%–70%), is observed in a temperature range from 210 °C to 410 °C; and can be associated primarily with the thermal decomposition of the organic phase [56]. In the range 600–1000 °C there is another small drop in mass (from 77% to 82%), which may be associated with combustion of the organic matrix [57].

There are no thermogravimetric analysis data available for carmine, but it is known that the C.I. Natural Red 4 molecule consists of anthraquinone and glucose. The first mass loss (10%) is caused by loss of water. According to [58], the first obvious peak in the TG curve for glucose pyrolysis occurred at a temperature of 239 °C. The maximum mass loss rate of glucose occurred at 301 °C. Further observed mass loss may be caused by thermal degradation of the aromatic part of the carmine structure [59].

Thermal stability measurements were also performed for selected dye/biopolymer hybrid materials. The mass loss profiles of these samples are similar to those obtained for the marine sponge spongin. The thermogravimetric curves, irrespective of the quantity of adsorbed dye, show mass loss caused by the transformations that occur as the temperature increases. However, as the amount of dye in the hybrid material increases, its thermal stability increases. The mass loss of the initial sample at 800 °C, for a product obtained after 1 min of adsorption contact time, was 47%; while for 15 min it was 42%, and for 60 min equals 24%. The increase in the stability of the hybrid material compared with the native adsorbent and with C.I. Natural Red 4 can be attributed to hydrogen bonds and electrostatic interactions formed between hydroxyl groups of the marine sponge spongin and the dye. The observed temperature peaks of mass loss in the TG curves for *H. communis* spongin, C.I. Natural Red 4 and the dye/adsorbent hybrid material made it possible to verify the difference between these compounds. This serves to confirm the effectiveness of the method for obtaining the new composite material.

### 2.12. SEM

**Figure 12:**
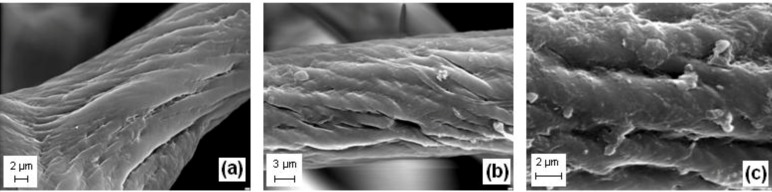
Scanning electron microscopy (SEM) micrographs of: (**a**) *H. communis* fiber (after demineralization); and (**b**,**c**) a selected dye/biopolymer hybrid material (50 mg/L, contact time 30 min, pH = 7) at different magnifications.

The SEM images in Figure 12 show the *H. communis* skeletal fibers and a selected dye/biopolymer hybrid material. Analysis of SEM images taken before and after adsorption confirmed that the deposition of C.I. Natural Red 4 onto the marine sponge skeletal fibers had taken place. The SEM images reveal the presence of dye microparticles (Figure 12b,c).

## 3. Experimental Section

### 3.1. Materials

Dried marine sponges of the species *Hippospongia communis* (Demospongiae) collected in Tunesian coastal waters were purchased from INTIB GmbH (Freiberg, Germany). The preparation of the adsorbent involved washing the dry sponge with fresh water to remove salts, and immersing it completely in 3 M HCl solution for 72 h at room temperature to dissolve foreign calcium carbonate—containing debris. The material was then rinsed with distilled water until the pH of the washing solution reached 6.5, and finally dried for 24 h at 50 °C in a drying oven.

C.I. Natural Red 4 in powder form was purchased from Sigma-Aldrich (St. Louis, MO, USA). The stock solution was prepared by dissolving an accurately weighed 500 mg portion of dye in 1000 mL of distilled water. Experimental solutions of desired concentration were obtained by successive dilutions with distilled water.

### 3.2. Adsorption and Desorption Experiments

Batch experiments (at 25 °C) were performed to investigate the effect of contact time, and to determine the kinetic parameters. Adsorption experiments were performed using 250 mL glass bottles containing 0.5 g of marine sponge skeleton as prepared above and 50 mL of the dye solution. The initial concentrations of the dye were 25, 50 and 75 mg/L, respectively.

After different time intervals the samples were filtered off under vacuum and taken for spectrophotometric evaluation (Spekol 1200, Analytik Jena, Jena, Germany) at the maximum absorbance wavelength 513 nm. Dye concentration in the adsorbent phase at a specific time (*q_t_*), and the adsorption efficiency (*E*%), were calculated as:

(5) $$q_{t}=\frac{(C_{0}-C_{t})\cdot V}{m}$$

(6) $$E\left(\%\right)=\frac{C_{0}-C_{t}}{C_{0}}\cdot 100\%$$

where *C*_0_ and *C_t_* are the concentrations of the dye in the solution before and after sorption respectively (mg/L), *V* is the volume of solution (L), and *m* is the mass of the support (g).

The effect of pH on the adsorption of carmine from aqueous solution onto the marine sponge skeleton was investigated in a similar manner. The pH was adjusted to 3, 5, 7 and 9 using either 1 M HCl or 1 M NaOH.

A desorption experiment was performed by placing 0.5 g of selected samples in a 250 mL conical flask with 50 mL of water and shaking at room temperature for 1 h. The desorption of C.I. Natural Red 4 from the hybrid material was measured by UV-Vis absorption, as described above.

Adsorption isotherms were obtained by placing the samples of 0.5 g of marine sponge skeleton in a series of flasks containing 50 mL of dye solution at the desired initial concentrations (50–1500 mg/L) at room temperature. Dye concentration after 60 min of phase contact time was measured spectrophotometrically at the maximum absorbance wavelengths. The quantity of dye adsorbed at equilibrium (*q*_e_), was calculated from Equation (7):

(7) $$q_{t}=\frac{(C_{0}-C_{t})\cdot V}{m}$$

where *C_0_* and *C*_e_ are the initial and equilibrium concentration of dye (mg/L), *V* is the volume of solution (L), and *m* is the mass of the support (g).

The experimental data were used to determine Freundlich and Langmuir adsorption isotherms.

### 3.3. Testing of Physicochemical Properties

FT-IR spectral analysis was performed using a Vertex 70 (Bruker, Bremen, Germany). The samples were analyzed in the form of tablets, made by pressing a mixture of anhydrous KBr (*ca.* 0.1 g) and 1 mg of the tested substance in a special steel ring, under a pressure of approximately 10 MPa. Analysis was performed over a wavenumber range of 400–4000 cm^−1^ (at a resolution of 0.5 cm^−1^, number of scans: 64).

X-ray photoelectron spectra were obtained with a UHV/XPS/AES System (SPECS) with a PHOIBOS 100 analyzer (SPECS, Berlin, Germany) and Mg Kα anode (1253.6 eV). The background line was determined by Shirley’s method. The selected reference line was C 1s 284.8 eV (C–C, C–H).

NMR analysis was performed using a DSX spectrometer (Bruker). A sample of about 100 mg was placed in a rotator, made of ZrO_2_, 4 mm in diameter, which enabled spinning of the sample. Centrifugation at the magic angle was performed at a spinning frequency of 8 kHz. ^13^C CP/MAS NMR (Cross Polarization Magic Angle Spinning Nuclear Magnetic Resonance) spectra were recorded at 100.63 MHz in a standard 4 mm MAS probe using a single pulse excitation with high power proton decoupling (pulse repetition 10 s, spinning speed 8 kHz).

Raman scattering spectra were investigated in the spectral range 100–3800 cm^−1^ (number of scans: 1024). The non-polarized Raman spectra were recorded in a back scattering geometry, using the inVia Renishaw micro-Raman system. The inVia Raman spectrometer (Renishaw, Wotton-under-Edge, UK) enabled the recording of Raman spectra with a spatial resolution of about 1 μm. The spectral resolution was 2 cm^−1^. The excitation light used was a laser operating at 785 nm. The laser beam was tightly focused on the sample surface through a Leica 50 × LWD (long working distance) microscope lens with numerical aperture (NA) equal to 0.5, producing a laser beam with a diameter of about 2 μm. To prevent any damage to the sample, the excitation power was fixed at about 5 mW. The position of the microscope lens was piezoelectrically controlled during measurement.

A thermogravimetric analyzer (TG/DTA/DSC, model Jupiter STA 449F3, Netzsch, (Selb, Germany) was used to investigate the effect of heat on the samples. Measurements were carried out under a flowing nitrogen atmosphere (10 cm^3^/min) at a heating rate of 10 °C/min over the temperature range 25–1000 °C, with an initial sample weight of approximately 5 mg.

The morphology and microstructure of the samples were studied using SEM images recorded from an EVO40 scanning electron microscope (Zeiss, Oberkochen, Germany). Before testing, the samples were coated with Au for a period of 5 sec using a Balzers PV205P coater (Oerlikon Balzers Coating AG, Balzers, Liechtenstein).

## 4. Conclusions

Marine spongin-based demosponges have unique physicochemical properties, and as such may have many practical applications. The process of adsorption of C.I. Natural Red 4 onto *H. communis* was found to depend on pH and time. When the initial dye concentration increases, the adsorption capacity at equilibrium increases, while the adsorption efficiency decreases. This indicates that initial dye concentration plays an important role in the adsorption of dyes. The experimental data correspond to a pseudo-second-order kinetic model of adsorption, which indicates that the rate-controlling stage of the process involves chemical adsorption.

For the measured spectra (FT-IR, Raman, ^13^C CP/MAS NMR, XPS) of the carmine/spongin hybrid material, only slight changes are observed relative to the spectra of the adsorbent. The lack of any significant changes suggests that there are no strong interactions between carmine and spongin. A possible interaction mechanism which may explain these observations is the formation of hydrogen bonds between the –OH and –COOH of the dye and the marine sponge skeleton. Moreover, the results obtained for adsorption at different pH values suggest the existence of additional interactions. The highest adsorption efficiency is observed at low pH values. In future work, further studies of adsorption will be undertaken to investigate other parameters that may affect this process. These will include temperature, quantity of biosorbent and ionic strength, as well as additional analyses; and will be carried out to confirm our assumptions.

The use of this novel C.I. Natural Red 4 dyed spongin skeleton for medical applications, including drug delivery, will also be studied in future experiments.
